# Axillary accessory breast cancer reconstructed by a thoracodorsal artery perforator flap: A case report

**DOI:** 10.1097/MD.0000000000033672

**Published:** 2023-05-12

**Authors:** Akiko Yumoto, Yuki Otsuki, Takashi Nuri, Erika Higashino, Kosei Kimura, Mitsuhiko Iwamoto, Koichi Ueda

**Affiliations:** a Department of Plastic and Reconstructive Surgery, Osaka Medical and Pharmaceutical University, Osaka, Japan; b Department of Breast and Endocrine Surgery, Osaka Medical and Pharmaceutical University, Osaka, Japan.

**Keywords:** accessory breast cancer, axillary reconstruction, case report, postoperative radiotherapy, scar contracture, thoracodorsal artery perforator flap

## Abstract

Primary accessory breast cancer is rare and most commonly occurs in the axilla. Due to its low incidence, few studies have discussed axillary reconstruction after accessory breast cancer resection. In the present report, we describe a patient who underwent axillary reconstruction with a thoracodorsal artery perforator (TAP) flap after resection, and reconstruction methods after resection of axillary accessory breast cancer are discussed based on current and previous reports.

**Patient concerns:**

A 60-year-old woman presented with a 7-year history of a gradually growing lump in the left axilla.

**Diagnosis:**

The patient was diagnosed with latent breast cancer, axillary lymph node metastasis, or carcinoma of the accessory axillary breast with axillary lymph node metastasis.

**Interventions:**

After preoperative chemotherapy, tumor resection and axillary lymph node dissection were performed, followed by immediate axillary reconstruction using a TAP flap. The patient received postoperative adjuvant endocrine and radiation therapy (50 Gy).

**Outcomes:**

No recurrence or metastasis was observed for 5 years postoperatively. The reconstructed axilla was not bulky, and scar contracture was not observed, with a full range of motion of the shoulder joint.

**Conclusion:**

We described a patient who underwent immediate TAP flap reconstruction after resection of accessory breast cancer and axillary lymph node dissection, followed by postoperative radiation, which could cause scar contracture. The patient was followed up for more than 5 years after the operation and radiation therapy, and the appearance of the axilla and range of motion of the shoulder were good despite postoperative radiation.

## 1. Introduction

Primary accessory breast cancer is rare, accounting for 0.3% to 0.6% of all breast cancers, and occurs most commonly in the axilla.^[1]^ Due of its low incidence, the diagnostic procedures and therapeutic management of accessory breast carcinoma are not well established, with no definitive guidelines,^[2]^ so that the treatment of primary accessory breast cancer is similar to that of primary breast cancer, supplemented with preoperative or postoperative chemotherapy, radiotherapy, and endocrine therapy.^[3]^ Furthermore, there are few reports on axillary reconstruction after accessory breast cancer resection. The defects after resection in most cases are probably closed by direct suture due to skin laxity, but excessive direct closure probably causes skin insufficiency of the axilla and scar contracture, which results in limitation of the range of motion of the shoulder joint. Furthermore, postoperative radiation therapy could be performed for accessory breast cancer, which may also worsen postoperative scar contracture. From the perspective of reconstructive surgery, cases with a wide defect of the axilla or postoperative radiation therapy should be reconstructed with a skin flap to maintain the shoulder joint range of motion. Various methods, including skin grafts, local flaps, and various axial pattern flaps^[4–9]^ of axillary reconstruction in post-burn contracture and resection of hidradenitis suppurativa have been reported, but the methods of reconstruction after axillary resection for accessory breast cancer have not been discussed in detail. In the present report, we describe a case of reconstruction using a thoracodorsal artery perforator flap after resection of a primary accessory breast cancer and postoperative radiation therapy. Five-year postoperative results are shown, and reconstructive methods after resection of the primary accessory breast cancer are discussed in the context of the current case and previous reports.

## 2. Case report

**Figure 1.**
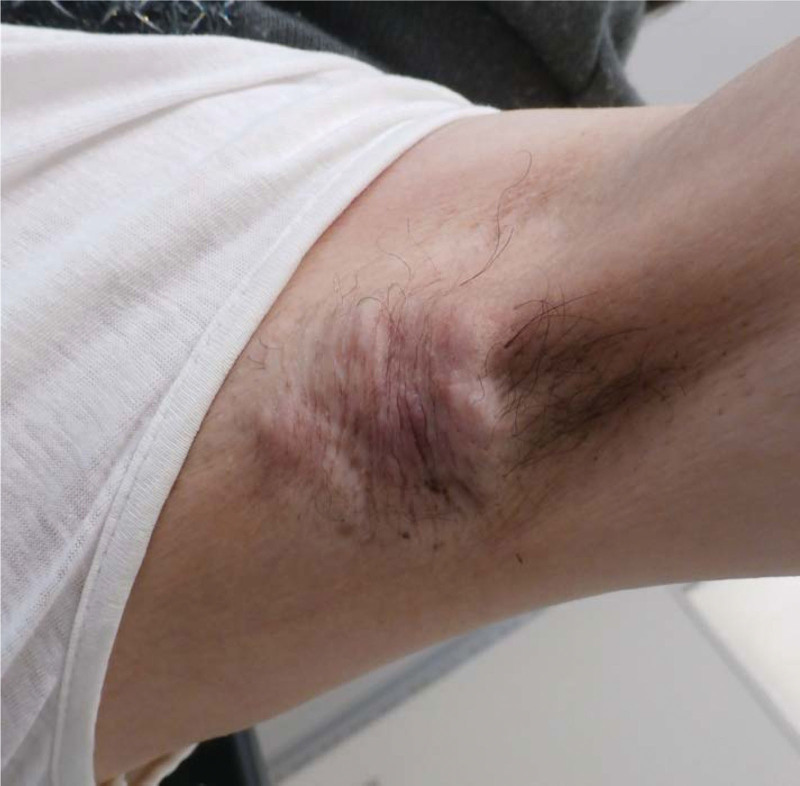
There was an irregularly shaped mass with skin erythema in the left axillary subcutaneous area measuring 50 × 35 mm^2^. They exhibit rigidity and poor mobility.

**Figure 2.**
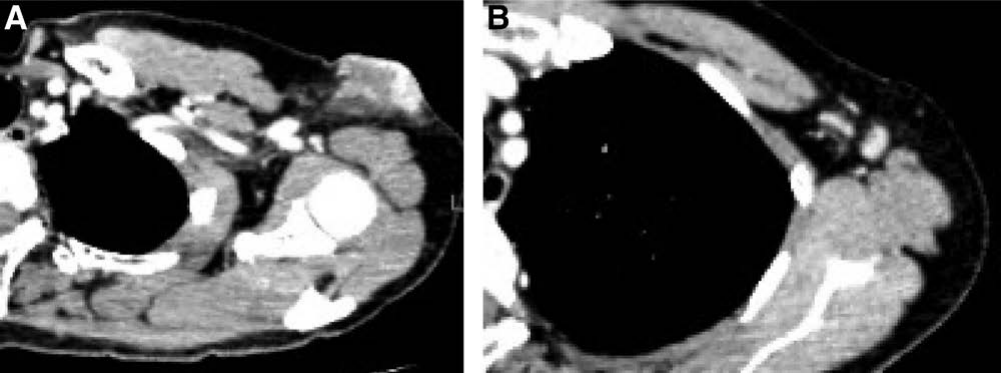
(A) A darkly stained mass, 41 × 25 mm^2^ in size, is seen under the skin of the left axilla to the subcutis. (B) The left axillary lymph nodes are enlarged.

A 60-year-old woman presented with a lump in the left axilla that had been gradually increasing in size for 7 years. She had no relevant medical history; however, her sister had breast cancer. On physical examination, a 50 × 35 mm^2^ firm, poorly mobile mass was palpated in the left axilla (Fig. 1). Computed tomography, magnetic resonance imaging, and positron emission tomography-computed tomography showed a 41 × 25 mm^2^ skin-contiguous mass in the left axillary subcutaneous tissue and surrounding lymphadenopathy apart from this tumor (Fig. 2). No abnormal findings were observed in the bilateral breasts or distant metastases. Trucut biopsy of the tumor and core needle biopsy of the axillary lymph node showed adenocarcinoma. Further routine immunohistochemical analyses were performed on formalin-fixed tissue, including estrogen receptor, progesterone receptor, human epidermal growth factor receptor 2, and Kiel67 antigen (Ki-67). Estrogen receptor was positive, progesterone receptor was negative, human epidermal growth factor receptor 2 was negative, and greater than 40% of cells were Ki-67-positive.

Based on these findings, the patient was diagnosed with latent breast cancer, axillary lymph node metastasis, or carcinoma in the accessory axillary breast with axillary lymph node metastasis. The oncological staging was stage IIIB (T4N1M0). She underwent preoperative chemotherapy (4 courses of *fluorouracil, epirubicin*, and *cyclophosphamide* and 4 courses of *docetaxel*), and the tumor and lymph nodes became much smaller. After therapy, tumor resection and axillary lymph node dissection were performed, followed by immediate axillary reconstruction with a thoracodorsal artery perforator flap (TAP flap).

### 2.1. Surgical findings

**Figure 3.**
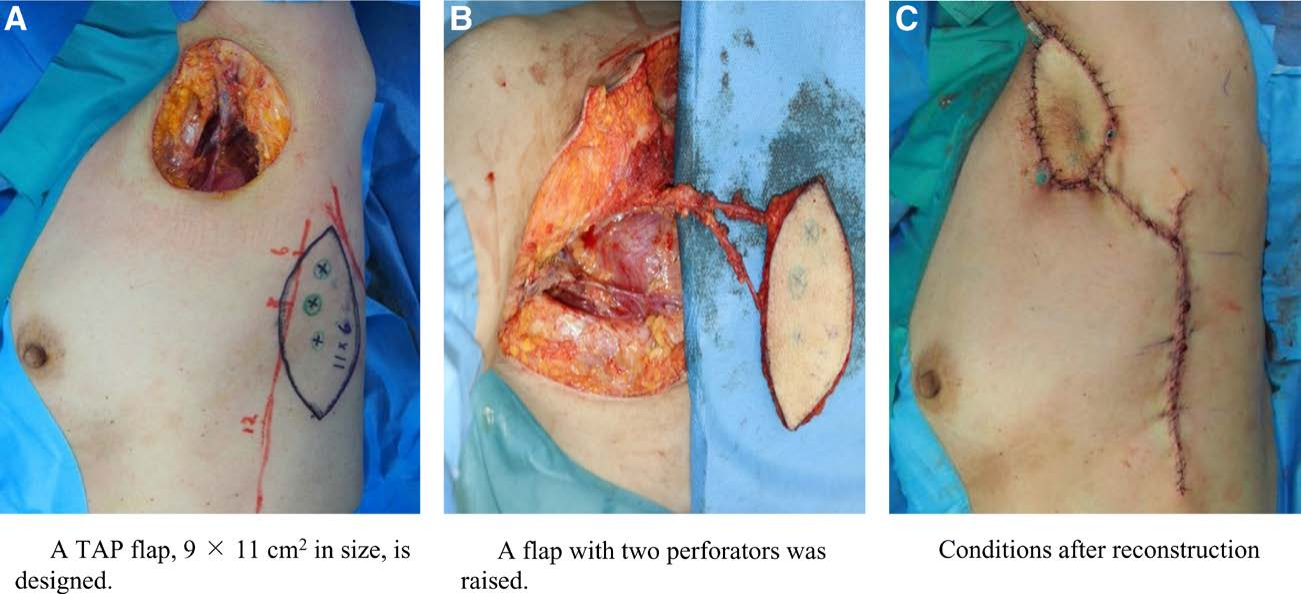
(A) A TAP flap, 9 × 11 cm^2^ in size, is designed. (B) A flap with 2 perforators was raised. (C) Conditions after reconstruction.

Tumor resection and lymph node dissection were performed simultaneously, resulting in a tissue defect measuring 8 × 5 cm^2^. A TAP flap 9 × 11 cm^2^ in size was designed, including 2 large perforating branches located 6 cm and 10 cm caudal to the caudal edge of the posterior axillary folds. A TAP flap was raised from the lateral side of the flap to detect the 2 perforators, which were then isolated proximally to the thoracodorsal artery. The TAP flap was rotated 180°clockwise to cover the axillary defect (Fig. 3).

Histopathological examination of the surgical specimen showed invasive ductal carcinoma and the surrounding normal mammary gland, and the diagnosis was axillary accessory breast carcinoma. The patient received postoperative adjuvant endocrine therapy (*letrozole*) 1 month after the operation, and postoperative radiation therapy (50 Gy/25 fractions) was administered to the left axilla and supraclavicular fossa.

**Figure 4.**
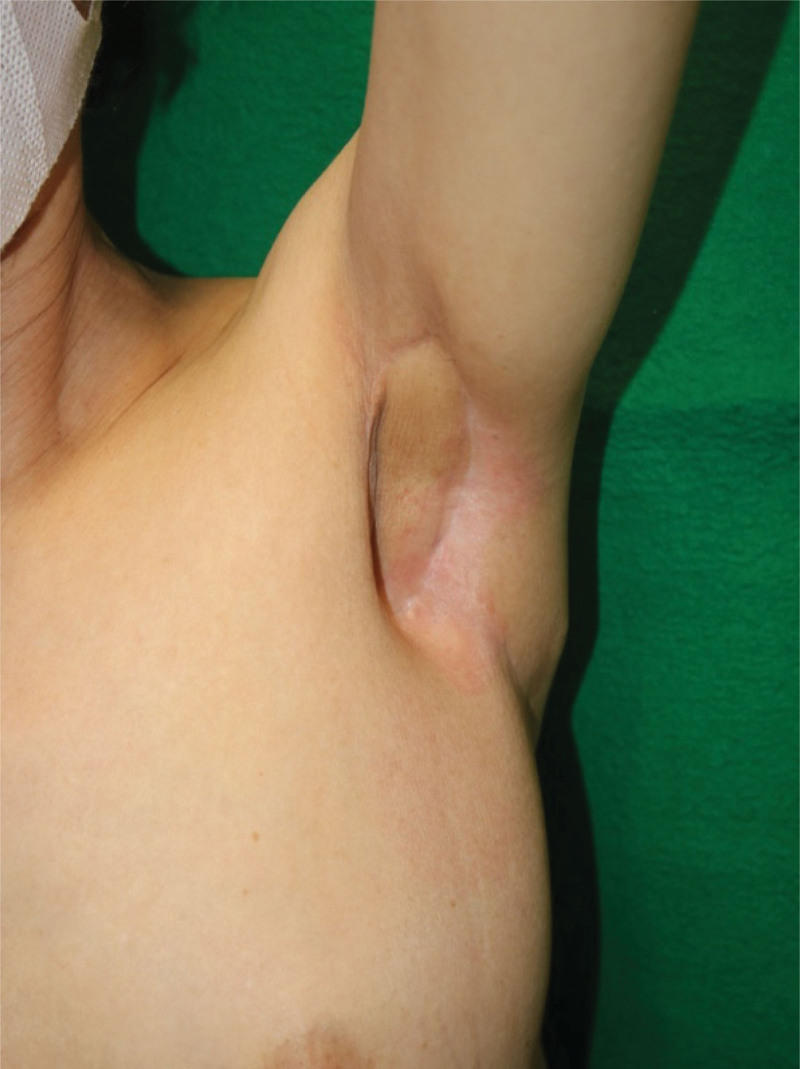
The range of motion of the shoulder is good, and the flap is not bulky and maintains good conformity.

No recurrence or metastasis was observed 5 years after surgery. Scar contracture was not observed, and her shoulder joint range of motion was full. In addition, the axilla was not bulky after surgery because the TAP flap was very thin and flexible (Fig. 4).

## 3. Discussion

Bilateral mammary ridges or milk lines run symmetrically along the ventral surface of the body from the anterior axillary fold to the medial aspect of the inguinal fold on both sides. They involute during embryogenesis, except in the pectoral region, to give rise to breast tissue, and persistence of the tissue can result in ectopic or accessory breast tissue.^[10]^ The prevalence of accessory breast tissue ranges from 2% to 6% in females and 1% to 3% in males. It is found more often in Asians than in Caucasians, with a high prevalence of approximately 5% in the Japanese population.^[11]^

The occurrence of malignancy in this ectopic breast tissue is extremely uncommon, constituting 0.3% to 0.6% of all breast cancer cases, generally occurring as axillary tumors.^[1]^ Due to its low incidence, the diagnostic procedures and therapeutic management of accessory breast carcinoma are not well established, with no definitive guidelines,^[2]^ so its treatment is performed according to that for breast cancer. Zhang et al^[3]^ hypothesized that when the tumor in the accessory breast is located close to the normal breast, it should be treated in the same manner as a cancer located in the normal breast. Accessory breast cancer is treated surgically and is supplemented with preoperative or postoperative chemotherapy, radiotherapy, and endocrine therapy.

The surgical reconstruction of axillary tissue defects remains controversial because of the complex surface of the axilla. The shoulder joint has the greatest range of motion in the human body, and restricted motion of the shoulder joint leads directly to decreased postoperative quality of life. Therefore, appropriate reconstruction is important. When flap reconstruction is planned for the axilla, the flap size should be larger than the defect size to prevent scar contracture, and a thin flap is preferred to avoid a bulky axilla.

Various reconstruction methods for axillary defects have been reported for advanced breast cancer, hidradenitis suppurativa, and post-burn scars.^[4–9]^ Although skin grafting is the simplest reconstruction procedure, it has several disadvantages. It is difficult to fit skin grafts into the concave surface of the axilla. It is necessary to keep the shoulder splinted in abduction to prevent skin grafts from wrinkling. Therefore, patients need to endure postoperative rest and rehabilitation, resulting in complicated postoperative management.^[5]^

Many locally pedicled myocutaneous flaps, such as latissimus dorsi myocutaneous flaps, pectoralis major myocutaneous flaps, and trapezius myocutaneous flaps, have been used as island flaps for axillary reconstruction.^[5]^ These flaps are easy to transfer to the axilla, but the main disadvantage of using myocutaneous flaps is their bulkiness. Bulkiness can limit the range of shoulder adduction, limiting movement.

Several cutaneous flaps vascularized from the subscapular artery branches, such as scapular/parascapular flaps, are more suitable for covering axillary defects. They provide thinner flaps and good texture and color matching. Because sacrificing muscles is minimized, several complications, such as seromas and hematomas, which occur more frequently with muscle resection, probably decrease. Furthermore, the donor site wound may be primarily closed without skin graft coverage, and is easily hidden in the posterior axillary fold and underwear.

A TAP flap is also widely used for axillary reconstruction because of the following attributes: basic reliable anatomy, the flap’s capacity to thin without compromising its blood supply, pliability of the flap, and lack of significant donor site morbidity.^[5–9]^ A TAP flap provides a longer pedicle than a scapular/parascapular flap, so that the flap can be rotated greatly and fit more easily to the defect.

Many reports on flap reconstruction, including TAP flaps for axillary defects, have been published for hidradenitis suppurativa and post-burn scars.^[5–9]^ However, there have been few reports of flap reconstruction for defects after axillary accessory breast cancer resection. To the best of our knowledge, Giuseppe et al reported a case of axillary accessory breast cancer reconstruction using a TAP flap designed according to Limberg.^[12]^ However, it showed the postoperative view of the flap 5 weeks after the operation, and it referred only to flap survival. Axillary reconstruction is important for preventing scar contracture and maintaining the range of motion of the shoulder joint. 5-week postoperative follow-up period was too short to show the outcome of flap reconstruction. In the present case, the patient underwent immediate TAP flap reconstruction after accessory breast cancer resection and postoperative radiation therapy. The patient was followed up for more than 5 years postoperatively, with no recurrence. Furthermore, postoperative photographs showed a good appearance of the axilla, and the range of motion of the shoulder was full despite the postoperative radiation. This was only a case study, and many cases are needed to evaluate the postoperative outcomes. However, a 5-year follow-up was sufficient to show the outcome of flap reconstruction, and it is important to share the results of the middle- or long-term follow-up following flap reconstruction of the axilla.

## 4. Conclusion

Axillary accessory breast cancers are rare. Treatment often requires axillary resection, as in breast cancer, and there are various reconstructive methods for axillary defects. A TAP flap is also widely used for axillary reconstruction, which provides a long pedicle so that it can be rotated greatly and fit more easily to the defect. In the present case, the patient underwent immediate TAP flap reconstruction after accessory breast cancer resection and axillary lymph node dissection, as well as postoperative radiation that could cause scar contracture. The patient was followed up for more than 5 years after the operation and radiation therapy, and the appearance of the axilla and the range of motion of the shoulder were good despite postoperative radiation.

## Author contributions

**Conceptualization:** Yuki Otsuki.

**Data curation:** Erika Higashino, Kosei Kimura, Mitsuhiko Iwamoto.

**Formal analysis:** Takashi Nuri.

**Supervision:** Yuki Otsuki, Koichi Ueda.

**Writing – original draft:** Akiko Yumoto.

**Writing – review & editing:** Yuki Otsuki, Takashi Nuri, Erika Higashino, Kosei Kimura, Mitsuhiko Iwamoto, Koichi Ueda.
